# Comparative Examination of Feline Coronavirus and Canine Coronavirus Effects on Extracellular Vesicles Acquired from A-72 Canine Fibrosarcoma Cell Line

**DOI:** 10.3390/vetsci12050477

**Published:** 2025-05-15

**Authors:** Sandani V. T. Wijerathne, Rachana Pandit, Chioma C. Ezeuko, Qiana L. Matthews

**Affiliations:** 1 Microbiology Program, Alabama State University, Montgomery, AL 36104, USA; sandani50828@gmail.com (S.V.T.W.); rachanapandit45@gmail.com (R.P.); cezeuko4096@myasu.alasu.edu (C.C.E.); 2 Department of Biological Sciences, College of Science, Technology, Engineering, and Mathematics, Alabama State University, Montgomery, AL 36104, USA

**Keywords:** A-72 cells, canine coronavirus, feline coronavirus, extracellular vesicles, exosomes, pathogenesis, immunomodulation

## Abstract

**Introduction:** Coronavirus (CoV) is an extremely contagious, enveloped positive-single-stranded RNA virus, which has become a global pandemic that causes several illnesses in humans and animals. Hence, it is necessary to investigate viral-induced reactions across diverse hosts. Herein, we propose utilizing naturally secreted extracellular vesicles (EVs), mainly focusing on exosomes to examine virus–host responses following CoV infection. Exosomes are small membrane-bound vesicles originating from the endosomal pathway, which play a pivotal role in intracellular communication and physiological and pathological processes. We suggested that CoV could impact EV formation, content, and diverse immune responses in vitro. **Methods:** In this study, we infected A-72, which is a canine fibroblast cell line, with a feline coronavirus (FCoV) and canine coronavirus (CCoV) independently in an exosome-free media at 0.001 multiplicity of infection (MOI), with incubation periods of 48 and 72 h. The cell viability was significantly downregulated with increased incubation time following FCoV and CCoV infection, which was identified by performing the 3-(4,5-dimethylthiazo-1-2yl)-2,5-diphenyltetrazolium bromide (MTT) assay. After the infection, EVs were isolated through ultracentrifugation, and the subsequent analysis involved quantifying and characterizing the purified EVs using various techniques. **Results:** NanoSight particle tracking analysis (NTA) verified that EV dimensions fell between 100 and 200 nm at both incubation periods. At both periods, total protein and RNA levels were significantly upregulated in A-72-derived EVs following FCoV and CCoV infections. However, total DNA levels were gradually upregulated with increased incubation time. Dot blot analysis indicated that the expression levels of ACE2, IL-1β, Flotillin-1, CD63, caspase-8, and Hsp90 were modified in A-72-derived EVs following both CoV infections. **Conclusions:** Our results indicated that FCoV and CCoV infections could modulate the EV production and content, which could play a role in the development of viral diseases. Investigating diverse animal CoV will provide in-depth insight into host exosome biology during CoV infection. Hence, our findings contribute to the comprehension and characterization of EVs in virus–host interactions during CoV infection.

## Simple Summary

Coronaviruses (CoVs) are a family of viruses that affect animals and humans. The interaction between companion animals and human host is of the utmost importance. Our research evaluates the impact of CoVs on extracellular vesicle production and composition in a canine cell line (A-72) following feline and canine coronavirus infections. The interplay of viruses and extracellular vesicles could be a means of viral pathogenesis within companion animal host.

## 1. Introduction

Severe acute respiratory syndrome coronavirus 2 (SARS-CoV-2) is an exceptionally contagious virus initially discovered in December 2019 in Wuhan, China [1,2]. The coronavirus disease 2019 (COVID-19), caused by SARS-CoV-2, is the most critical global health crisis since the 1918 influenza outbreak [3,4]. CoVs belong to the family Coronaviridae, and these viruses are enveloped, single-stranded (ss) RNA viruses mainly transmitted via respiratory droplets [5,6]. CoVs cause numerous diseases, such as respiratory, neurological, gastrointestinal, and liver diseases [7,8,9]. This virus can infect diverse hosts, including humans, felines, canines, cows, pigs, and birds [5,10]. SARS-CoV and Middle East respiratory syndrome coronavirus (MERS-CoV) originated from bats, and these viruses pose potential health risks to humans [11]. Evidence suggests that felines and canines, like companion animals, are vulnerable to infections caused by animal and human coronavirus (HCoVs) [12,13]. FCoV and CCoV are extremely contagious single-stranded RNA viruses found in cats and dogs, respectively [14]. FCoV was initially detected in 1960, and it was an asymptomatic infection primarily found in the gastrointestinal tract of domestic felines [15,16]. Enteric FCoV (FECV) and Feline Infectious Peritonitis (FIP) are the two primary biotypes of FCoV [5]. FECV commonly results in mild or subclinical intestinal diseases, but in some felines, it can mutate and cause FIP [5,17]. FIP is a fetal illness defined by acute inflammation, immune imbalance, and dysfunction of various organs [18]. Therefore, it remains a primary reason for mortality among young felines globally, emphasizing the severe pathogenetic capability [19]. CCoV was first identified in 1970, and it typically causes mild gastroenteritis in dogs [20,21]. However, in some cases, CCoV can spread beyond the intestine. This can cause widespread diseases influencing other organs like the kidney, liver, and pulmonary system [22]. These systemic diseases mainly occur when the canine is co-infected with other microorganisms or when the virus strains are specifically virulent [21,22,23]. CCoV is classified into two distinct genotypes, CCoV type I and CCoV type II, both of which belong to the Alphacoronavirus genus [24,25]. Both viruses are primarily spread through the fecal–oral route and are closely related genetically [14,26,27]. Furthermore, several reports indicated that dogs and cats are susceptible to being infected with SARS-CoV-2, FCoV, and CCoV [13,28,29]. Studies have shown that recombinant alphacoronavirus from feline and canine is present in humans undergoing severe respiratory syndromes, which indicates the significant risk of medical conditions in humans caused by FCoV and CCoV [14,30]. Hence, it is pivotal to investigate the inter-species specificity, tissue tropism, and susceptibility of FCoV and CCoV that directly interact with humans to evade their transmission to humans. SARS-CoV-2 enters the host cells mainly when the coronavirus spike (S) protein attaches to the angiotensin-converting enzyme 2 (ACE2) receptor, discovered on the surface of the host cells [31,32].

Extracellular vesicles (EVs) have become notable interests in viral infection investigations, particularly concerning their function in pulmonary diseases and inflammation [33,34]. EVs can enclose viral particles, proteins, and RNA; this process eases the transmission of the virus to healthy cells and improves virus reproduction [35,36]. They are small, membrane-enclosed nanoparticles released from various cell categories into external space and found in blood, saliva, breast milk, serum, and urine [37,38]. EVs are crucial in intercellular communication, immune modulation, disease biomarkers, directed therapeutic delivery, and gene therapy [5,39,40]. Primarily based on EV formation, they are classified into three subtypes: apoptotic bodies, microvesicles, and exosomes [41,42]. This research specifically studies the exosome’s host–pathogen responses after CoV infection. Exosomes are produced from the internal budding of initial-phase endosomes into multivesicular bodies (MVBs) [43]. Then, MVBs fuse with the cell membrane to expel exosomes [44,45]. This process facilitates exosomes to specifically encapsulate an assortment of bioactive molecules such as micro-RNA, RNA, DNA, proteins, and lipids, and then transfer them to target cells [46,47,48]. Hence, exosomes play a crucial role in cell-to-cell signaling, affecting numerous pathological and physiological mechanisms [46,49]. Therefore, exosomes are the most investigated EV category. EVs play a vital role in increasing viral infectiousness and spread, as shown by Hepatitis C virus (HCV)-infected cells releasing envelope glycoprotein-coated exosomes for the purpose of evading antibody neutralization, and Coxsackieviruses of group B using infected receptor-negative host cells, thus enabling the transmission of these viruses via innovative mechanisms [36,50,51]. Additionally, EVs are essential in modulating immune responses during viral infections in Human Immunodeficiency Virus (HIV) infections; Nef-containing exosomes derived from contaminated cells interfere with cholesterol metabolism in healthy cells, resulting in systemic inflammation and HIV-related comorbidities [52,53,54]. Moreover, Epstein-Barr virus, Hepatitis viruses, and HIV have been demonstrated to exploit exosomal microRNAs to regulate host immune responses, inhibit antiviral mechanisms, and facilitate their persistence and proliferation [55,56,57]. The literature has reported that EVs released from SARS-CoV-2-infected pulmonary cells transported viral RNA and cellular receptors to cardiac cells [58,59]. This insight illustrated the function of EVs in easing viral spread and affecting the disease progression of COVID-19 [58]. Additionally, studies have suggested that circulating plasma-derived exosomes with ACE-2 from disease-free individual and convalescent COVID-19 patients have the ability to inhibit the attaching viral S protein to surface binding sites [60,61,62,63]. This finding indicated that EVs could facilitate and potentially control the transmission of the COVID-19 virus. Moreover, EVs can facilitate viral proliferation by inhibiting the body’s innate and adaptive immunological defenses [35,64]. Therefore, numerous viral illnesses have identified this extensively researched mechanism of EV encapsulation, release, and delivery between cells while regulating the immune responses throughout the infectious phase and assisting the uninfected cells’ viral transmission.

In this research, we hypothesized that feline and canine CoVs could exploit the exosomal trafficking route and impact EV formation and content within the host cells. We intend to study the distinctive impact of CCoV and FCoV infection on the formation and content of A-72-derived EVs. A-72 is a fibroblast cell line that demonstrates adherence properties and primarily originates from a tumor in a Golden Retriever dog’s thigh [65,66]. A-72 is susceptible to both FCoV and CCoV. Our results illustrated that CCoV induces more substantial modifications in A-72-derived EV formation and content than FCoV. Moreover, EVs released from A-72 cells after independent infection with FCoV and CCoV at 0.001 MOI are time-dependent and result in CCoV-infected EV’s elevated expression of crucial biomarkers compared to FCoV-infected EVs. These results implied that CCoV has an enhanced ability to use EVs for its immune modulation, viral transmission, stress response activation, and clearance relative to the FCoV. In further investigation, our objective is to examine the receptor-independent entry pathways and extracellular virion formation of FCoV and CCoV in the host cells. Therefore, demonstrating the distinct mechanisms of immune regulation and viral dissemination between FCoV and CCoV enables pivotal insights into the pathogenesis of CoVs, which could help to prevent emerging CoV outbreaks.

## 2. Methods

### 2.1. Cell Culture

The A-72 adherent fibroblast cell line was utilized as the host model for FCoV and CCoV and was acquired from the American Type Culture Collection (ATCC) [67]. A-72 cells were cultured in Roswell Park Memorial Institute Medium (RPMI, Fisher Scientific, Grand Island, NY, USA) with L-glutamine supplemented with 10% Corning regular fetal bovine serum (FBS, Fisher Scientific, Grand Island, NY, USA), 1% penicillin/streptomycin (Fisher Scientific, Grand Island, NY, USA), and 0.2% amphotericin B (Fisher Scientific, Grand Island, NY, USA). A-72 cells were cultured until the confluency of 70–80% with a 5% CO_2_-containing incubator at 37 °C. For FCoV and CCoV infections, exosome-depleted media were prepared using RMPI supplemented with 2% exosome-depleted Corning FBS following the laboratory guidelines.

### 2.2. Viral Stock

Feline coronavirus (Strain: WSU791683(3), Serotype I, FECV biotype) and canine coronavirus (Strain: 1–71) stocks were obtained from ATCC. The final concentrations of the acquired FCoV and CCoV viral stocks were 8.9 × 10^3^ TCID_50_/mL and 1.6 × 10^6^ TCID_50_/mL, respectively. The essential multiplicity of infection (MOI) for both FCoV and CCoV infections was ascertained using an infection cycle for 10 days by monitoring the cytopathic effect (CPE).

### 2.3. In Vitro Infection of A-72 Cells with FCoV and CCoV

Once A-72 cells gained the confluency of 70–80%, the cells were trypsinized and counted using a countess cell counter (Invitrogen, Waltham, MA, USA) to determine the cell viability. Subsequently, A-72 cells (5.0 × 10^5^) were plated in a 60 mm × 15 mm cell culture dish, incubated overnight, and maintained under the required conditions (37 °C and 5% CO_2_). Afterward, the cell supernatant was removed, and 3 mL of RPMI supplemented with 2% exosome-depleted FBS was added to each dish. Control dishes were incubated after adding exo-depleted media. However, infection dishes were infected with FCoV and CCoV independently at 0.001 MOI for 48 h and 72 h time intervals. After 48 h and 72 h post-infection, the cell supernatant was obtained separately and stored at −80 °C until exosome isolation was conducted via ultracentrifugation. Every experimental condition (control and FCoV-infected, CCoV-infected) was conducted in 5 biological replicates.

### 2.4. Cell Lysates

The A-72 cells from each control and CoVs-infected cell culture dishes were collected by adding 1 mL of 1× phosphate-buffered saline (PBS) and kept for 1 min. The A-72 cells with PBS were centrifuged to gather cell pellets. After that, controls, FCoV-infected, and CCoV-infected cell lysates were made utilizing 400 µL of 1X lysis buffer prepared from the cell culture lysate 5X reagent (Promega, Madison, WI, USA) and stored at −80 °C for further experiments.

### 2.5. Isolation and Purification of A-72-Derived EVs Using Ultracentrifugation

Previously uninfected control, FCoV, and CCoV-infected cell supernatant were centrifuged for 10 min at 300× *g* at 4 °C utilizing the Allegra X-14R Centrifuge, Beckman Coulter. Subsequently, the cell supernatant was gathered again and further processed by spinning at 2600× *g* for a duration of 10 min and filtered via a 0.22 µm filtration membrane. Then, the filtered upper phase was conveyed into an ultracentrifuge tube, and residual volume was filled using 1X PBS and subjected to a further round of centrifugation utilizing a Beckman Coulter Optima L-70K ultracentrifuge for 45 min at 20,000× *g* (4 °C). Then, the supernatant was centrifuged again, and centrifugation was conducted for 70 min at 160,000× *g*. Finally, 500 μL of purified control and CoV-infected EVs were gathered, and protease inhibitor (Thermo Scientific, Waltham, MA, USA) was added prior to storing the EVs at −80 °C for continued investigations [5,68].

### 2.6. Evaluate the A-72 Cell Viability Utilizing the MTT (3-(4,5-Dimethylthiazo-1-2yl)-2,5-diphenyltetrazolium Bromide) Assay

The MTT assay was used to examine the cell viability and cytotoxicity. A-72 cells (1 × 10^4^) were plated in triplicate separately in 96-well plates. Subsequently, cells were incubated overnight under the required conditions (37 °C and 5% CO_2_). The next day, cell-free media were removed, and exo-depleted RMPI media were added. The infection wells were infected with FCoV and CCoV, independently, at 0.001 MOI for 48 h and 72 h time intervals. However, the control wells were incubated, subsequently adding exo-depleted media. Then, 50 μL of 5 mg/mL MTT in 1X PBS was introduced to A-72 cells and incubated for 4 h. Finally, absorbance was measured at 570 nm in a microplate reader.

### 2.7. Assessment of EV Sizes and Concentrations Utilizing NanoSight Tracking Analysis (NTA)

NTA was conducted to examine the concentration and nanoparticle size of A-72-derived control, FCoV-infected, and CCoV-infected EVs. NTA is a technique for visualizing and analyzing the size of the particle in fluids according to the rate of Brownian motion to dynamic light scattering (DLS). The EV samples were diluted with 1X PBS (1:100) before being injected into the ZetaVIEW R Particle Tracking Analyzer’s chamber. For this assay, the mean values were examined in 11 different locations.

### 2.8. Quantitation of Total Protein After FCoV and CCoV Infections

A Bicinchoninic Acid (BCA) Assay was used to measure the total quantity of exosomal and lysate proteins. A range of standard concentrations (bovine serum albumin) and EV and lysate samples from A-72-derived control, FCoV-infected, and CCoV-infected conditions, each at a concentration of 5 µg/µL, were introduced to a 96-well plate in triplicate. Then, protein reagents A (25 µL) and B (200 µL) were introduced to each well, and the plate was positioned on a shaker wrapped with aluminum foil. The absorbance was measured at 595 nm using a microplate reader after 10 min to identify the precise total protein concentration in A-72 isolated EVs via plotting a standard curve.

### 2.9. Analysis of Total RNA/DNA After FCoV and CCoV Infections

The total RNA and DNA were extracted from both A-72-CoV-derived exosomes and lysate utilizing the TRIzol solution (Thermo Fisher, Waltham, MA, USA). For DNA and RNA extraction, EV and lysate samples at a concentration of 5 µg/µL from control, FCoV-infected, and CCoV-infected conditions were introduced to RNase-free DNase I treatment and micrococcal nuclease (MNase) treatment, respectively. Prior to the MNase treatment, A-72 isolated EVs and lysate samples were incubated on ice for 30 min with 1% Triton-X-100. For total DNA analysis, EV and lysate samples were introduced to Ethylenediaminetetraacetic acid (EDTA) treatment at 65 °C (10 min). Then, the TRIzol method was followed for DNA and RNA extraction [5,69,70]. Finally, Nanodrop was utilized to examine EV and lysate total DNA and RNA levels from both A-72-CoV-derived exosomes and lysates.

### 2.10. Dot Blot Analysis

Dot blot analysis was conducted to quantify the composition of proteins correlated with A-72-CoVs-derived exosomes. Approximately 5 µg of A-72-derived control, FCoV-infected, and CCoV-infected EVs were treated with reducing buffer and placed on a heating block at 95 °C for 10 min. Subsequently, the EV samples were dotted on a nitrocellulose membrane and blocked utilizing 5% non-fat dry milk made in 1X Tris-buffered saline (TBS), including 0.2% Tween-20 for 35 min. Afterward, the nitrocellulose membrane was washed 3 times using TBST buffer for 10 min and incubated overnight at 4 °C with primary antibodies (1:250–1:1000) directed against exosome-specific markers, membrane trafficking proteins, coronavirus-specific proteins, immune response-specific proteins, stress-specific proteins, and apoptosis proteins. The following day, the membrane was washed three times with TBST buffer before introducing secondary antibodies (1:500–1:2000), which can be either horseradish peroxidase (HRP)-conjugated goat anti-mouse, anti-rabbit, or anti-rat antibodies in blocking buffer for 1–2 h at room temperature (RT). The particular protein signals were detected employing SuperSignal West Femto Maximum Sensitivity Substrate (Invitrogen, Waltham, MA, USA), and the image was processed employing the Bio-Rad ChemiDoc™ XRS+ System (Bio-Rad Laboratories, Hercules, CA, USA).

### 2.11. Statistical Analysis

Statistical analysis was executed employing a one-way analysis of variance (ANOVA) with Tukey post hoc analysis for multiple group analyses of the collected data utilizing GraphPad, Version 5. Statistical significance is represented by the mean ± standard deviation (SD) as listed in *p* ≤ 0.05 (*), *p* ≤ 0.01 (**), *p* ≤ 0.001 (***), and *p* ≤ 0.0001 (****).

## 3. Results

### 3.1. A-72 Cell Viability After FCoV and CCoV Infections

**Figure 1:**
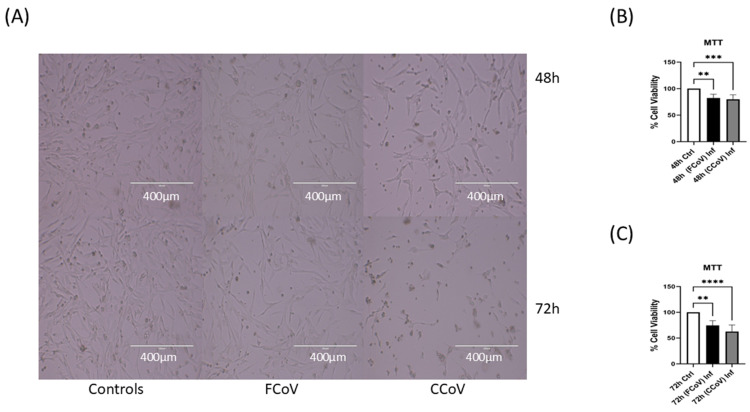
Canine fibrosarcoma cell line (A-72), cell viability following FCoV and CCoV infection. (**A**) Bright-field microscopy images demonstrated A-72 cellular morphology at 48 h and 72 h time intervals. (**B**) A-72 cells were infected independently with FCoV and CCoV in exo-depleted RMPI media at 0.001 MOI at 48 h and (**C**) 72 h. Following the incubations, A-72 cells were incubated again with MTT assay dye for 4 h (37 °C), and absorbance was detected at 570 nm. Statistical analysis was executed utilizing a one-way analysis of variance (ANOVA) with Tukey post hoc analysis for collected data values. Statistical significance is exhibited by the mean ± standard deviation (SD) as listed in *p* ≤ 0.01 (**), *p* ≤ 0.001 (***), and *p* ≤ 0.0001 (****).

A-72 cells were infected independently with FCoV and CCoV at 0.001 MOI and examined at 48 h and 72 h post-infection time intervals. The morphology of A-72 cells was determined utilizing bright-field microscopy, demonstrating a lowered quantity of cells with an extended incubation period for both viruses; CCoV lowered the number of viable A-72 cells to a greater extent than FCoV, which indicated that CCoV infection led to more efficient hijacking of cellular processes relative to FCoV, resulting in heightened cellular demise (Figure 1A). The MTT cell viability analysis indicated a substantial downregulation in the viability of A-72 cells with an extended incubation period after infection. Figure 1B indicated that the viability of cells was significantly downregulated at 48 h, nearly 18% (** *p* ≤ 0.01) and 20% (*** *p* ≤ 0.001) FCoV and CCoV, respectively, in comparison to control A-72 cells. Figure 1C indicated that the viability of cells was significantly downregulated at 72 h time interval, nearly 25% (** *p* ≤ 0.01) and 37% (**** *p* ≤ 0.0001) FCoV and CCoV, respectively, relative to control A-72 cells. These results illustrated that both FCoV and CCoV infection led to notable cytopathic effects and increased cell death in A-72 cells relative to control CRFK cells at 48 and 72 h time points.

### 3.2. FCoV and CCoV Modified A-72-Derived EV Particle Size and Concentration

**Figure 2:**
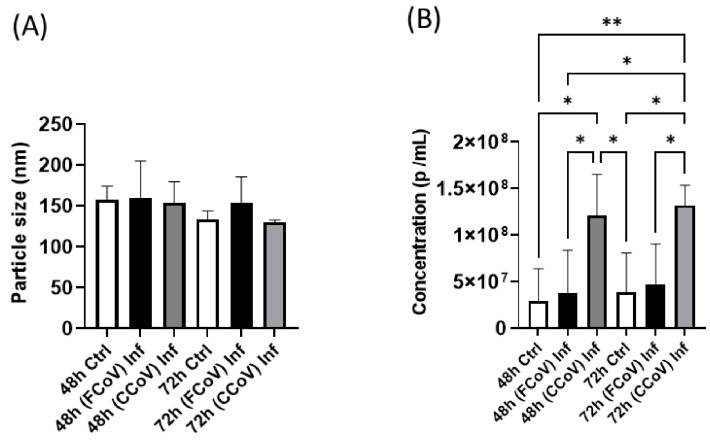
Characterization of A-72-originated EVs after FCoV and CCoV infection. (**A**) NTA analysis illustrated mean particle size distribution; (**B**) particle concentration distribution following FCoV and CCoV infection at 48 h and 72 h time markers. Statistical analysis was executed utilizing a one-way ANOVA with Tukey post hoc analysis. Statistical significance is exhibited by the mean ± SD as listed in *p* ≤ 0.05 (*) and *p* ≤ 0.01 (**).

NTA was conducted to investigate the EV nanoparticle size range and density in an aqueous solution. These insights are crucial for comprehending the biophysical characteristics of A-72-derived EVs. These A-72-derived EV properties are pivotal in FCoV and CCoV infection pathogenesis and disease development by facilitating cell signaling via carrying host and viral biological molecules between cells. Figure 2A indicates that the mean particle size profile of FCoV-exposed EVs and CCoV-exposed EVs was slightly increased (160.2 nm) and slightly decreased (153.6 nm), respectively, compared to control EVs (157.6 nm) from A-72 cells at 48 h. Similarly, at the 72 h time interval, the mean particle size profile of FCoV-exposed EVs and CCoV-exposed EVs originating from A-72 cells was slightly increased (154.4 nm) and decreased (130.1 nm), respectively, in comparison to control EVs (134.2 nm) originating from A-72 cells. However, these differences in EV size were not statistically significant. Figure 2B shows that CCoV-exposed A-72-derived EV concentration (particles/mL) was significantly increased (1.2 × 10^8^; 1.3 × 10^8^) as compared to both FCoV-exposed EVs (3.7 × 10^7^; 4.7 × 10^7^) and control EVs (2.9 × 10^7^; 3.8 × 10^7^) at 48 h and 72 h, respectively. Therefore, higher CCoV-exposed EV concentration indicated that CCoV may trigger stronger EV-facilitated responses and elevate viral spread than FCoV.

### 3.3. A-72 Produced EVs and Lysates Biomolecule Content Following Both FCoV and CCoV Infections

**Figure 3:**
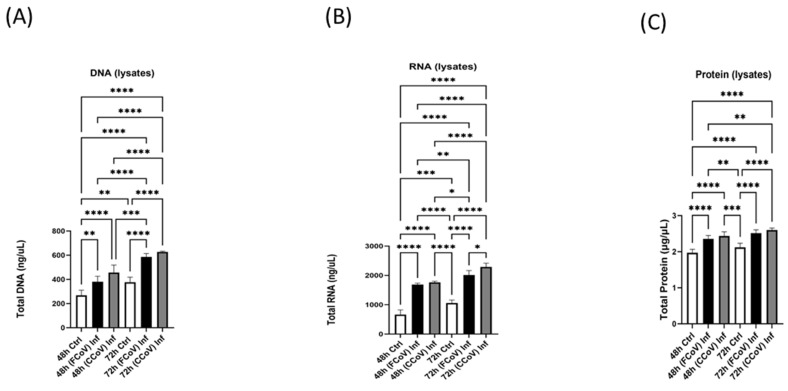
The biomolecular content of FCoV-exposed and CCoV-exposed lysates. (**A**) total DNA; (**B**) total RNA; and (**C**) total protein concentration of post-FCoV and CCoV infection lysates at 48 h and 72 h time intervals. Statistical analysis was executed utilizing a one-way ANOVA with Tukey post hoc analysis. Statistical significance is exhibited by the mean ± SD as listed in *p* ≤ 0.05 (*), *p* ≤ 0.01 (**), *p* ≤ 0.001 (***), and *p* ≤ 0.0001 (****).

**Figure 4:**
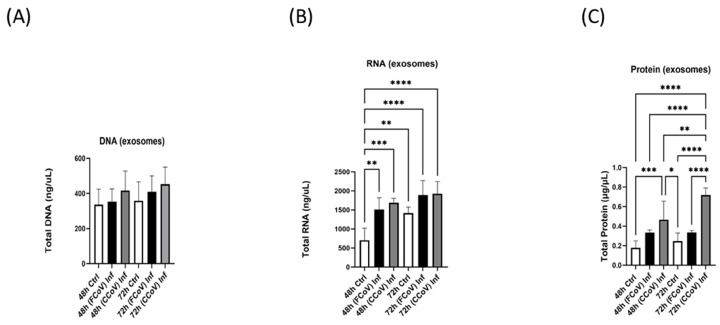
The biological relevance of FCoV-exposed and CCoV-exposed A-72-originated EVs. (**A**) total DNA; (**B**) total RNA; and (**C**) total protein concentration of A-72-derived EVs after FCoV and CCoV infection at 48 h and 72 h time intervals. Statistical analysis was executed utilizing a one-way ANOVA with Tukey post hoc analysis. Statistical significance is exhibited by the mean ± SD as listed in *p* ≤ 0.05 (*), *p* ≤ 0.01 (**), *p* ≤ 0.001 (***), and *p* ≤ 0.0001 (****).

Analyzing the total DNA, RNA, and protein concentration aids in discovering the viral influence on cellular mechanisms. The total concentration of DNA (Figure 3A and Figure 4A), RNA (Figure 3B and Figure 4B), and protein (Figure 3C and Figure 4C) were determined in the control lysates and EVs, FCoV-infected lysates and EVs, and CCoV-infected lysates and EVs at 48 h and 72 h time intervals. Cell lysates offer insights into cellular composition, such as DNA, RNA, and proteins after infections. A significant increase in DNA concentration was observed between lysates of FCoV- and CCoV-infected cells compared to the control cells at both 48 h (** *p* ≤ 0.01, **** *p* ≤ 0.0001) and 72 h (**** *p* ≤ 0.0001) post-infection (Figure 3A). Figure 3B indicated the total RNA in lysates, confirming that the FCoV-infected and CCoV-infected lysates were significantly increased compared to control lysates at 48 h (**** *p* ≤ 0.0001 and **** *p* ≤ 0.0001, respectively). At the 72 h time interval, FCoV-infected lysates were significantly increased relative to the control lysates (**** *p* ≤ 0.0001). Additionally, at the 72 h time point, CCoV-infected lysates were significantly increased when compared to the FCoV-infected lysates (* *p* ≤ 0.05) and control lysates (**** *p* ≤ 0.0001). Figure 3C shows that the total lysate protein concentrations of FCoV-exposed and CCoV-infected lysates were significantly increased compared to control lysates at 48 h (**** *p* ≤ 0.0001 and **** *p* ≤ 0.0001, respectively) and 72 h (**** *p* ≤ 0.0001 and **** *p* ≤ 0.0001, respectively) post-infection.

Figure 4A shows a negligible increase in total EV DNA concentration with extended incubation duration after FCoV and CCoV infection. Figure 4B indicated that total RNA concentration in FCoV-infected EVs (** *p* ≤ 0.01) and CCoV-infected EVs (*** *p* ≤ 0.001) was significantly increased when compared to the A-72-derived control EVs at a 48 h time interval. Figure 4C illustrated that total protein concentration in CCoV-infected EVs originating from A-72 cells was significantly increased (*** *p* ≤ 0.001) compared to control EVs at 48 h. Additionally, at 72 h, total protein concentration of CCoV-infected EVs was significantly increased relative to FCoV-infected EVs (**** *p* ≤ 0.0001) and control EVs (**** *p* ≤ 0.0001).

### 3.4. Expression of Classical Exosome Protein Elevated in Response to FCoV and CCoV Infection

**Figure 5:**
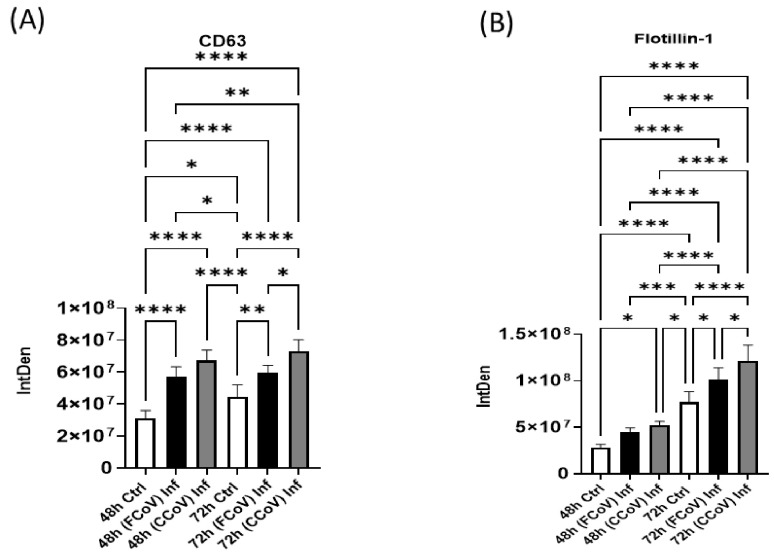
The presence of classical exosome protein and membrane trafficking protein following FCoV and CCoV infection. Graphs demonstrate the quantitative densitometry examination of dot blot analysis of (**A**) CD63 after 48 h and 72 h infections, (**B**) flotillin-1 after 48 h and 72 h infections in control, and FCoV-infected and CCoV-infected EVs isolated from A-72 cells. Statistical analysis was executed utilizing a one-way ANOVA with Tukey post hoc analysis. Statistical significance is exhibited by the mean ± SD as listed in *p* ≤ 0.05 (*), *p* ≤ 0.01 (**), *p* ≤ 0.001 (***), and *p* ≤ 0.0001 (****).

Cluster of Differentiation 63 (CD63) is a classical exosome biomarker, a tetraspanin protein [71]. CD63 is commonly used in research applications as an objective for extracting and characterizing exosomes [72]. In the present study, we performed a dot blot analysis to examine the expression of CD63 in A-72-derived control EVs, FCoV-infected EVs, and CCoV-infected EVs at 48 h and 72 h time intervals. The levels of CD63 were significantly increased in FCoV- and CCoV-infected A-72-derived EVs when compared to the control EVs at 48 h (**** *p* ≤ 0.0001 and **** *p* ≤ 0.0001, respectively) (Figure 5A). Moreover, Figure 5A illustrates that the FCoV-infected EVs exhibited a substantially elevated level of CD63 at 72 h as compared to the control EVs (** *p* ≤ 0.01). We identified that CD63 was significantly upregulated in CCoV-exposed EVs at the 72 h time marker relative to the FCoV-infected EVs (* *p* ≤ 0.05) and control EVs (**** *p* ≤ 0.0001). Therefore, CD63 expression was higher in CCoV-infected EVs than in FCoV-infected EVs, indicating distinct levels between the two infections.

### 3.5. Presence of Cellular Membrane Trafficking Protein After FCoV and CCoV Infection

Flotillin-1 is a membrane-trafficking biomolecule vital in diverse cellular processes, including membrane transport and cell signaling [73]. In this study, we identify the level of Flotillin-1 in A-72-derived control EVs, FCoV-infected EVs, and CCoV-infected EVs. We found that Flotillin-1 was significantly upregulated after CCoV infection at 48 h (* *p* ≤ 0.05) (Figure 5B). Additionally, Flotillin-1 was found to be significantly elevated in FCoV-infected EVs at 72 h, relative to the control EVs (* *p* ≤ 0.05). Flotillin-1 expression was significantly increased in CCoV-infected EVs at 72 h in comparison to the FCoV-infected EVs (* *p* ≤ 0.05) and control EVs (**** *p* ≤ 0.0001). These findings suggested that CCoV has a more noticeable influence on Flotillin-1 compared to the exposure to FCoV at 48 h and 72 h time intervals, highlighting that CCoV has greater efficacy in influencing cellular processes associated with membrane trafficking.

### 3.6. Evaluation of Host Receptor After FCoV and CCoV Infection

**Figure 6:**
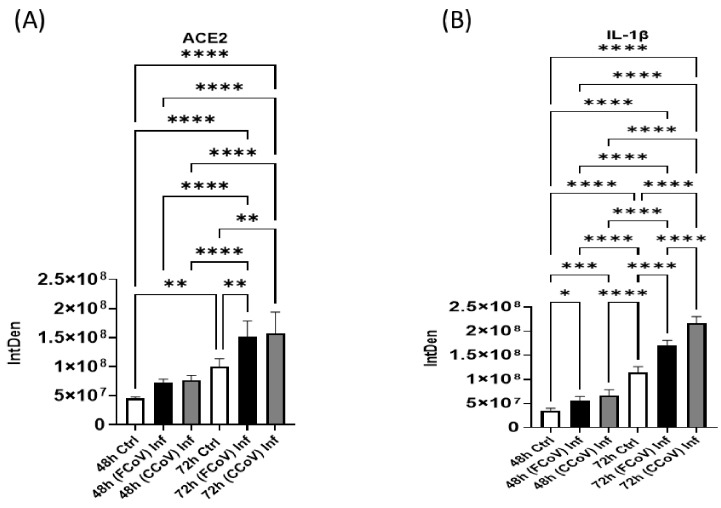
The impact of FCoV and CCoV infections on host receptor protein and pro-inflammatory response. Graphs demonstrate the quantitative densitometry examination of dot blot analysis of (**A**) ACE2 after 48 h and 72 h infections, (**B**) IL-1β after 48 h and 72 h infections in control, and FCoV-infected and CCoV-infected EVs isolated from A-72 cells. Statistical analysis was executed utilizing a one-way ANOVA with Tukey post hoc analysis. Statistical significance is exhibited by the mean ± SD as listed in *p* ≤ 0.05 (*), *p* ≤ 0.01 (**), *p* ≤ 0.001 (***), and *p* ≤ 0.0001 (****).

Angiotensin-converting enzyme 2 (ACE2) is pivotal in numerous physiological processes [74]. ACE2 is the primary cell receptor for SARS-CoV and SARS-CoV-2 ingress into the host cells [5,75]. Hence, ACE2 has been widely researched concerning the CoV outbreak due to its function in aiding viral penetration and following disease development [31,76]. The spike (S) protein consists of two main subunits, S1 and S2 [77]. SARS-CoV-2 S protein is essential to viral infiltration by binding to ACE2 [75,78]. Current findings indicate that attaching the S protein of SARS-CoV-2 to ACE2 is able to increase ACE2 enzyme function [79]. Figure 6A revealed that ACE2 was substantially elevated in FCoV-exposed (** *p* ≤ 0.01) and CCoV-exposed (** *p* ≤ 0.01) EVs originating from A-72 cells at 72 h in comparison to control EVs. Therefore, our findings suggested that the existence of ACE2 receptors in FCoV- and CCoV-exposed EVs originated from A-72 cells. Importantly, both FCoV- and CCoV-infected EVs showed closely similar expression levels of ACE2. Hence, this confirmed that both FCoV and CCoV could use comparable mechanisms to modulate ACE2 levels in A-72 cells.

### 3.7. Expression of Pro-Inflammatory Response Post-Infection with FCoV and CCoV

We examined the Interleukin-1 beta (IL)-1β in EVs purified from A-72 after FCoV and CCoV infections. IL-1β is a pro-inflammatory marker in the body’s inflammatory reaction [80]. IL-1β is generated mainly via stimulated dendritic cells, monocytes, and macrophages [81]. IL-1β is engaged in the modulation of immunological responses and the development of various inflammatory disorders [82,83]. FCoV- and CCoV-infected A-72 cells’ EVs induced a substantial rise in IL-1β protein content relative to the control EVs at 48 h (* *p* ≤ 0.05 and *** *p* ≤ 0.001, respectively) (Figure 6B). IL-1β was substantially upregulated in FCoV-exposed EVs at 72 h time intervals compared to the control EVs (**** *p* ≤ 0.0001). Furthermore, IL-1β was significantly increased in CCoV-infected EVs at 72 h as compared to FCoV-infected EVs (**** *p* ≤ 0.0001) and control EVs (**** *p* ≤ 0.0001). Therefore, our results confirm that CCoV exhibited significantly upregulated expression levels of IL-1β compared to FCoV, indicating that FCoV stimulates pro-inflammatory responses less effectively than CCoV.

### 3.8. Stress Response Biomarker Expression Elevated in Response to FCoV and CCoV Infections

**Figure 7:**
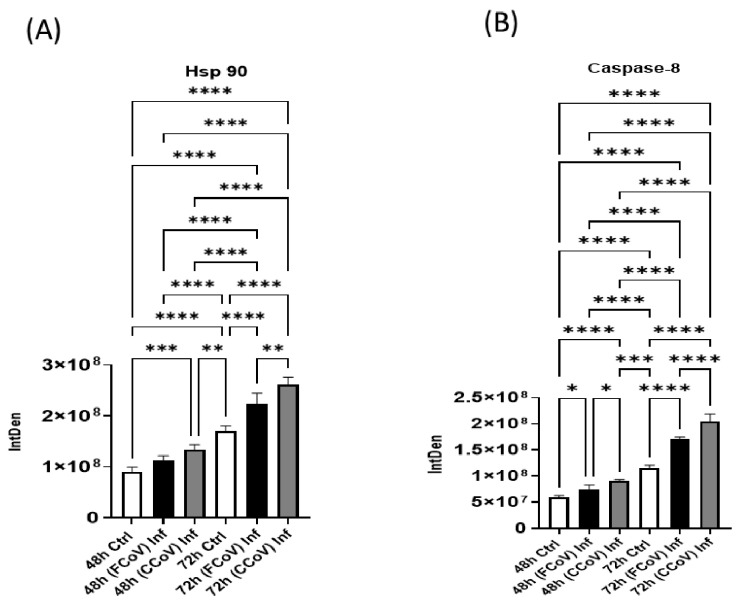
The impact of FCoV and CCoV infections on heat shock protein and caspase. Graphs demonstrate the quantitative densitometry examination of dot blot analysis of (**A**) Hsp90 after 48 h and 72 h infections, (**B**) caspase-8 after 48 h and 72 h infections in control, and FCoV-infected and CCoV-infected EVs isolated from A-72 cells. Statistical analysis was executed utilizing a one-way ANOVA with Tukey post hoc analysis. Statistical significance is exhibited by the mean ± SD as listed in *p* ≤ 0.05 (*), *p* ≤ 0.01 (**), *p* ≤ 0.001 (***), and *p* ≤ 0.0001 (****).

Heat shock proteins (Hsps) are vital in preserving cellular balance and serve as molecular chaperones [84]. Hsps are elevated as a reaction to numerous stressors, such as inflammatory responses, heat, and oxidative damage [85]. They facilitate the accurate folding of newly formed proteins, inhibit protein aggregation, and refold the malformed proteins [84,86]. We evaluated the purified EVs for the presence of Hsp90, which functions to maintain and trigger a wide range of target proteins [87,88]. Figure 7A illustrates that Hsp90 was substantially elevated in CCoV-infected EVs compared to the uninfected control A-72-derived EVs at 48 h (*** *p* ≤ 0.001). Moreover, Hsp90 was significantly enhanced in FCoV-infected EVs at 72 h compared to A-72-derived control EVs (**** *p* ≤ 0.0001). Furthermore, at the 72 h time marker, Hsp90 was significantly upregulated in CCoV-infected EVs relative to the FCoV-infected EVs (** *p* ≤ 0.01) and uninfected control EVs (**** *p* ≤ 0.0001). Hence, these results confirmed that CCoV has a more significant effect on cellular stress processes and EV composition than FCoV. Additionally, the distinct level of Hsps in response to FCoV and CCoV infections may offer a crucial understanding regarding their pathogenesis.

### 3.9. FCoV and CCoV Infections Regulate Apoptotic Activation

In this study, we evaluated the levels of caspase-8, a cysteine protease that is a vital enzyme in the cell death pathway [89]. Caspase-8 is the primary initiator in the extrinsic program cell death pathway, and it plays a crucial role in Nuclear Factor kappa beta (NF-κβ) activation [89,90,91]. Figure 7B indicated that caspase-8 was significantly upregulated in FCoV-exposed EVs compared to the control EVs at 48 h (* *p* ≤ 0.05). Additionally, caspase-8 was significantly elevated in CCoV-exposed EVs relative to the FCoV-exposed EVs and control EVs at 48 h (* *p* ≤ 0.05 and **** *p* ≤ 0.0001, respectively). We found that caspase-8 was significantly elevated in FCoV-exposed EVs at 72 h compared to the control EVs (**** *p* ≤ 0.0001). Furthermore, CCoV-exposed EVs exhibited a significantly upregulated level of caspase-8 at 72 h time marker in comparison to the FCoV-infected EVs (**** *p* ≤ 0.0001) and control EVs (**** *p* ≤ 0.0001). These findings proposed the differential effects of FCoV and CCoV on caspase-8 presence in A-72-derived EVs. These results confirm that CCoV has a more significant impact on caspase-8 regulation in EVs compared to the FCoV. This difference in caspase-8 levels could indicate different virus–host interactions and cellular reactions to both FCoV and CCoV.

## 4. Discussion

EVs play a crucial role in the disease mechanism and development of viral infections [5,64,92,93]. They assist in cell-to-cell communication by carrying viral components and host biological molecules, including DNA, RNA, proteins, and lipids [46,94,95]. This process can elevate viral replication and propagation [35]. By activating inflammation, EVs can regulate the immune reaction mechanism [96,97]. Researchers have also investigated EVs concerning viral infections such as influenza, HPV, and HIV, where they aid in disease development by regulating immune system responses and promoting virus spread [35,98,99]. Furthermore, exosomes have demonstrated potential effectiveness in treating COVID-19. For example, exosomes obtained from mesenchymal stem cells (MSCs) have the capacity to reduce inflammation and cytokine storms in individuals infected with COVID-19 [100,101]. Hence, further research is necessary to comprehend EV’s clinical application and effectiveness in managing human CoVs, animal CoVs, and other viral infections.

In this current investigation, we assessed the effect of FCoV and CCoV infection on A-72-derived EV formation and composition. A-72 cells were infected with FCoV and CCoV independently at 0.001 MOI and incubated for 48 h and 72 h time intervals. This is the first study of this kind comparing the impacts of coronavirus infections on EVs in the same cell line. After incubation, the microscopic analysis demonstrated a reduced quantity of viable cells in CCoV-exposed A-72 cells relative to FCoV-exposed A-72 cells with extended incubation time, confirming CCoV’s stronger cell-damaging effect compared to FCoV (Figure 1A). Additionally, the MTT assay indicated that CCoV-infected A-72 cells have more pronounced downregulation in cell viability relative to the FCoV-infected A-72 cells as time progresses (Figure 1B,C). The elevated cytotoxicity and greater reduction in cellular survival observed with CCoV infection relative to FCoV infection confirmed stronger virulence. NTA analysis illustrated that the isolated control EVs, FCoV-infected EVs, and CCoV-infected EVs fall within the size range typically linked with the exosomes, which range from 30 to 200 nm. (Figure 2A). Particle concentration was significantly increased in CCoV-exposed EVs compared to both uninfected control EVs and FCoV-exposed EVs at both time points, which indicated that CCoV infection has a more significant impact on EV production from A-72 cells than FCoV (Figure 2B). Additionally, the detected increases in DNA, RNA, and protein content in CCoV-infected lysates and EVs indicate increased cellular function and EV-associated molecular production relative to FCoV infection. This could suggest that CCoV induces a stronger EV response. However, this finding might also be affected by the inherent distinctions in reproduction behavior between FCoV and CCoV. Our results confirm the contribution of both FCoV and CCoV in regulating EV formation and cargo composition in A-72 cells.

In this research, we analyzed the presence of various protein indicators, such as classical exosome biomarker (CD63), membrane transport molecule (Flotillin-1), virus target host receptor marker (ACE2), immune marker (IL-1β), stress-related marker (Hsp90), and apoptotic response indicator (caspase-8) in control EVs obtained from A-72 cells, FCoV-infected EVs and CCoV-infected EVs at 48 h and 72 h time intervals. We verified that CD63 significantly increased in CCoV-infected EVs compared to both FCoV-infected and control EVs at 48 h and 72 h time markers (Figure 5A). CD63 is a tetraspanin protein broadly acknowledged as an indicator for exosomes and plays important functions, including cellular attachment, immune system modulation, and movement [5,102,103,104]. CD63 is found on the surface of EVs, and can facilitate exosome production indirectly by suppressing its internalization, resulting in its aggregation at the cell membrane and enhanced exosome formation [105,106]. Additionally, CD63 has the capacity to function as a biomarker for virus-induced diseases [107,108]. For example, in HIV-1, CD63 has been associated with the emission of viral components, and in SARS-CoV-2, CD63 engages in virus packaging and immune system interaction [109,110]. CCoV infection may enhance the EV release dynamics, which may assist the viral spread and impact host immune reactions compared to FCoV. Increased CD63 expression in CCoV-infected EVs could further assist in immune-associated communication, as CD63 is linked with EV-mediated immunological regulation in viral infections. Additionally, these findings emphasize the unique pathogenesis mechanisms between FCoV and CCoV infection. The expression of Flotillin-1 in CCoV-exposed EVs was substantially elevated compared to FCoV-exposed EVs and uninfected EVs at 72 h (Figure 5B). Flotillin-1 has the capability to link with lipid rafts and generate scaffolding structures, rendering it essential for modulating several cellular functions [5,111,112]. Its dysfunction has been associated with diverse disease states, such as neurodegenerative illnesses and cancer [111]. In HIV-1 and SARS-CoV-2, Flotillin-1 assists in viral formation and membrane fusion [113]. Flotillin-1 plays a vital role in membrane trafficking, signal transmission, and endocytosis [5,73,111]. Therefore, CCoV can increase vesicular sorting and EV secretion more proficiently compared to FCoV, highlighting particular mechanisms of protein transport and assembly within the lipid raft, which can aid viral disease progression.

The existing literature has reported that ACE2 function is the main entry receptor for SARS-CoV-2, which enables viral penetration to target cells and significantly contributes to COVID-19 disease [31,75,76,114]. ACE2 can be found in numerous tissues, such as renal organs, pulmonary organs, heart, and digestive pathways, which clarifies the multi-system influence of COVID-19 [76,115]. Modulation of ACE2 expression throughout the viral diseases could emphasize its capacity as a treatment objective [116,117,118]. Additionally, the interplay between ACE2 and EVs can highlight how EVs could carry ACE2 and impact viral disease development [117,119]. We determined that ACE2 was significantly increased in FCoV-infected and CCoV-infected EVs relative to control EVs at the 72 h time point (Figure 6A). Additionally, both viruses indicated similar expression levels of ACE2 in A-72-originated EVs at both time intervals. Hence, FCoV and CCoV could use similar processes to impact ACE2 presentation in A-72 cells. Although ACE2 is not a recognized receptor for FCoV or CCoV, its existence in EVs during these infections could indicate a broader function in viral disease development and cell-to-cell communication. ACE2 has been demonstrated to be enclosed in EVs in other coronavirus infections, like SARS-CoV-2, where ACE2 may regulate immune reactions or act as a possible co-receptor. Hence, assessing ACE2 in EVs from FCoV- and CCoV-infected cells could offer knowledge regarding preserved mechanisms of EV-mediated host–virus interactions across various coronaviruses.

Additionally, IL-1β is a pro-inflammatory cytokine crucial in inflammatory responses during SARS-CoV-2 and zika virus infections [120,121,122]. IL-1β is a primary mediator of tissue destruction in numerous inflammatory disorders [80,123]. Moreover, it stimulates the expression of Interleukin-6 (IL-6) and Tumor Necrosis Factor-alpha (TNF-α)-like pro-inflammatory cytokines [124,125,126]. IL-1β was significantly upregulated in CCoV-infected EVs relative to both FCoV-infected and control EVs (Figure 6B). These findings suggested that CCoV could more successfully utilize an inflammatory route to facilitate disease development and increase EV release compared to FCoV. Moreover, Hsp90 is a protein chaperone that can maintain and stimulate various substrate proteins, especially in stressful conditions [88,127]. HSPs assist viral reproduction and stability in various viruses such as HIV-1 and SARS-CoV-2 [128]. The elevated expression level of Hsp90 (Figure 7A) in CCoV infection compared to FCoV infection in an increased infection period demonstrated that there is a specific disease progression mechanism between FCoV and CCoV, where CCoV leads to more significant changes in EV content, which can aid the virus persistence and replication. Furthermore, caspase-8 is vital in apoptosis and immune responses [129,130]. COVID-19 investigations have revealed that caspase-8 levels are substantially elevated, which confirms that the virus activates the programmed cell death mechanisms, resulting in the demise of COVID-19 virus-infected cells [131,132]. Results showed substantially elevated levels of caspase-8 in CCoV-exposed EVs relative to both FCoV-exposed and uninfected control EVs at both time intervals (Figure 7B). These results indicated that CCoV infection might trigger more apoptosis-related signals and inflammatory reactions than FCoV. This confirms that caspase-8 activation in CCoV may enable virus multiplication and spread by promoting cellular demise.

The literature has stated the possibility of utilizing Crandell-Rees Feline Kidney (CRFK) cells as a model for investigating animal coronaviruses, specifically FCoV and CCoV; these studies provide deeper comprehension regarding distinct cell lines displaying differing responses to viral diseases due to their distinctive cellular properties [5,133]. A-72 is a canine cell line that can offer more suitable conditions for CCoV infection, possibly increasing viral pathogenesis relative to FCoV. This is mainly because of the CCoV and cell surface proteins present on A-72 cells that are pertinent for CCoV infection, which could aid more effective viral infiltration and replication. Although FCoV and CCoV are closely related, variations in EV reactions could indicate differences in viral reproduction, host interactions, or immune regulation that might impact their disease outcomes. Current examination illustrated a notable rise in all protein indicators and biomolecular analysis in A-72-derived EVs at 48 h relative to 72 h, which implies that prolonged incubation time increases the formation of EVs, secretion of these EVs, viral propagation, and linked cellular reactions. As a result, it influences EV composition and possible function on viral pathogenesis. Additionally, these results provided a comprehensive understanding of the different disease development mechanisms between FCoV and CCoV infections. Moreover, the elevated levels of protein indicators at 72 h compared to the 48 h time interval may suggest that increased viral disease development, immune activity regulation, cellular stress, and apoptotic responses offer an in-depth understanding of the dynamics of infection and possible therapeutic opportunities for CoV treatments.

## 5. Conclusions

EVs are obtaining a notable interest in viral infection investigations because of their functions in disease pathogenesis, immune regulation, and possible therapeutic interventions. They can aid cell signaling by carrying viral and host biological molecules such as DNA, RNA, and proteins, impacting viral propagation and immune responses. EVs have the potential to carry cargo across extended distances in the body, which can impact distant cellular structures and host organs. Therefore, this systemic transport is able to increase virus dissemination and survival. Our research verified that A-72-originating EVs infected with CCoV in vitro significantly influence cell viability, host cellular functions, cellular responses, cargo content, EV production, and release as compared to FCoV infection. Our comparative examination showed that CCoV infection led to a significant elevation of EV-associated protein markers relative to FCoV infection. Particularly, we identified increased expression of classical exosome markers, pro-inflammatory proteins, membrane trafficking molecules, stress-specific proteins, and caspase proteins in CCoV-infected EVs. Additionally, the coronavirus cellular receptor ACE2 shows comparable expression levels between FCoV- and CCoV-infected EVs compared to control EVs. While these molecular changes were clearly observed, their biological importance necessitates further examination. In future research, we will investigate the biological impacts of this EV modification through in vivo experiments to determine their function in viral pathogenesis, which could provide insights regarding the functions of EVs in viral diseases and their possible treatment target to manage future diseases resulting from companion animal CoVs.

## Data Availability

The original contributions presented in this study are included in the article. Further inquiries can be directed to the corresponding author(s).
